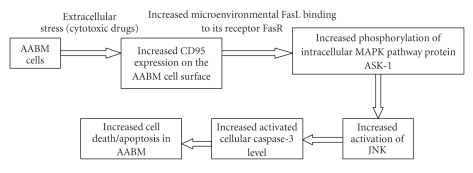# Alteration in Marrow Stromal Microenvironment and Apoptosis Mechanisms Involved in Aplastic Anemia: An Animal Model to Study the Possible Disease Pathology

**DOI:** 10.4061/2010/932354

**Published:** 2010-09-19

**Authors:** Sumanta Chatterjee, Ranjan Kumar Dutta, Pratima Basak, Prosun Das, Madhurima Das, Jacintha Archana Pereira, Malay Chaklader, Samaresh Chaudhuri, Sujata Law

**Affiliations:** Stem Cell Research and Application Unit, Department of Biochemistry and Medical Biotechnology, Calcutta School of Tropical Medicine, Calcutta 700073, India

## Abstract

Aplastic anemia (AA) is a heterogeneous disorder of bone marrow failure syndrome. Suggested mechanisms include a primary stem cell deficiency or defect, a secondary stem cell defect due to abnormal regulation between cell death and differentiation, or a deficient microenvironment. In this study, we have tried to investigate the alterations in hematopoietic microenvironment and underlying mechanisms involved in such alterations in an animal model of drug induced AA. We presented the results of studying long term marrow culture, marrow ultra-structure, marrow adherent and hematopoietic progenitor cell colony formation, flowcytometric analysis of marrow stem and stromal progenitor populations and apoptosis mechanism involved in aplastic anemia. The AA marrow showed impairment in cellular proliferation and maturation and failed to generate a functional stromal microenvironment even after 19 days of culture. Ultra-structural analysis showed a degenerated and deformed marrow cellular association in AA. Colony forming units (CFUs) were also severely reduced in AA. Significantly decreased marrow stem and stromal progenitor population with subsequently increased expression levels of both the extracellular and intracellular apoptosis inducer markers in the AA marrow cells essentially pointed towards the defective hematopoiesis; moreover, a deficient and apoptotic microenvironment and the microenvironmental components might have played the important role in the possible pathogenesis of AA.

## 1. Introduction

Aplastic anemia (AA) is an acquired disease characterized by an extremely hypocellular marrow and peripheral blood pancytopenia due to chronic depression of hematopoiesis in the bone marrow [1–3]. The exact causes and mechanisms involved in the bone marrow failure in aplastic anemia is still not quite steadily explained; however, it is clear that acquired aplastic anemia is a heterogeneous disease caused by different pathophysiological conditions [4, 5]. The possible pathophysiological conditions that account for AA include decreased hematopoietic stem cell (HSC) number, impaired hematopoietic stem cell function, and increased bone marrow cellular apoptosis level and the functional and structural defects in the bone marrow hematopoietic microenvironment and several microenvironmental components [6–9]. 

Hematopoietic stem/progenitor cell renewal and growth have long been discussed to be under the control of various cytokines and growth factors released by the marrow hematopoietic microenvironment [10]. Within the marrow cavity the mystery of stem/progenitor cell health has been found to be critically dependent on microenvironmental components which are of varied and diverse nature [11–13]. Recent studies have revealed that the hematopoietic bone marrow microenvironment is heterogeneous in respect to bone lining osteoblasts, fibroblasts, multilaminar and branched stromal barrier cells, and the reticuloendothelial cells [14, 15]. Indeed, the normal hematopoiesis requires a complex interplay between the hematopoietic cells and the marrow microenvironment which is necessary for switching on several proliferation and differentiation signaling cascade [16–18]. One of the hallmarks of aplastic anemia is the deficient functioning of the hematopoietic system and the hematopoietic microenvironment. Several studies have shown that the number of primitive hematopoietic progenitors (capable of hematopoiesis in long-term marrow cultures) is drastically reduced in the vast majority of patients with AA. Furthermore, when long-term marrow cultures (LTMC) were established from the aplastic anemic bone marrow, the proliferation of hematopoietic progenitors have been found to sustain only for few days and that also at very low levels. Thus, it is clear that severe quantitative and qualitative alterations exist within the compartment of hematopoietic stem/progenitor cells and in some cases, also within the hematopoietic microenvironment in the disease AA [19–22]. 

 Apoptosis (programmed cell death) is increased in several hematological disorders characterized by bone marrow failure. The fine equilibrium between the differentiation and apoptosis of normal hematopoietic cells get altered in AA. The exposure to nonphysiological programmed cell death could deregulate this equilibrium, resulting in excessive and uncontrolled apoptosis of hematopoietic cells in AA [23–27]. Indeed, it has been suggested that this mechanism of unregulated cell death is the cause of poor production of hematopoietic cells and an ineffective hematopoiesis in AA [28]. Furthermore, the normal bone marrow cells require certain viability factors, produced by the marrow microenvironment, to remain viable and undergo apoptosis when these factors are withdrawn. Thus, the reduced viability factors production by the marrow microenvironment can also be a contributing problem to the proliferation defect and/or the excessive apoptosis of the bone marrow cells in AA [29–33].

 The objective of the present study was to investigate the quantitative and qualitative alterations in the hematopoietic stem/progenitor cell compartment, bone marrow stromal hematopoietic microenvironment and several microenvironmental components in an animal model of drug-induced aplastic anemia. The objective further included the study of bone marrow cells from aplastic anemic mice in terms of clonogenic functional potential and the apoptosis signaling pattern in AA. The following four approaches were used: (1) analysis of the marrow microenvironment through long term bone marrow explant culture (LTBMC-Ex) system, scanning electron micrographs of the marrow ultrastructure and the colony forming efficacy of the marrow adherent stromal cells, (2) determination of the deviation in the clonogenic potential of the bone marrow derived cells in the aplastic anemia compared to the normal controls through semisolid colony forming assay, (3) flowcytometric characterization of the stem (Sca1^+^c-kit^+^) and stromal (Sca1^+^CD44^+^) progenitor cell population; (4) evaluation of the bone marrow cellular apoptosis level with subsequent alterations in the expression profile of several extracellular apoptosis marker (CD95/APO-1) and intracellular (pASK-1, pJNK, Cleaved Caspase-3) apoptosis inducer proteins under the event of the disease AA. On the basis of the above-mentioned observations, here, we have reported alterations in the physiological status of the stromal microenvironment in AA. Impaired proliferation, differentiation and increased cellular apoptosis in the aplastic anemic bone marrow have also been revealed. The present paper hoped to provide new insights in better understanding the underlying mechanisms that are involved in the initiation and progression of the disease AA.

## 2. Materials and Methods

### 2.1. Animals and Disease Induction

Inbred swiss albino mice of mixed sexes for both the normal control (*n* = 20) and experimental (*n* = 20) groups were housed in controlled room temperature of 25°C in the filter top cages on normal diet and water ad libitum. Twenty weeks old mice weighed to approximately 20–22 gms received 20 mg/kg body weight busulfan and 80 mg/kg body weight cyclophosphamide intraperitoneally on day 0th and 28th, respectively. Controls received comparable volumes of saline [34–37].

### 2.2. Blood Hemogram Profile

Twelve weeks after the second injection approximately 200–300 *μ*L of blood was collected by tail vein puncture from each of the experimental and normal control groups of animals using heparinized vials. The hemoglobin concentration, white blood cells (WBCs), red blood cells (RBCs) counts, platelets, reticulocytes, and neutrophils counts were determined using standard laboratory techniques.

### 2.3. Isolation of Bone Marrow

Twelve weeks after the second injection those experimental group of mice which showed progressive disease status were sacrificed to isolate the long bones (femur and tibia). Bone marrow was flushed out from the above-mentioned bones by a syringe containing RPMI-1640 media supplemented with 10% fetal bovine serum (FBS). Some portions of the isolated marrow were cut into small pieces (approximately 0.2 mm^3^) and kept in media containing FBS for further culture protocol and for preparations of the sample for scanning electron microscopy. The rest of the bone marrow was mixed well with repeated pipetting and passed through cell strainer to isolate single cells. The isolated single cells were then depleted of RBCs using RBC-depletion buffer (BD-Biosciences, USA). Finally the cells were kept in RPMI-1640 media supplemented with FBS.

### 2.4. Long-Term Bone Marrow Culture

Isolated small 0.2 mm^3^ fragments of the bone marrow explants were subjected to culture in triplicate (for each of the normal and experimental groups) in 75 mm culture dish (Corning, USA) containing 4 mL of RPMI-1640 supplemented with 30% FBS. The culture medium also consisted of 1% bovine serum albumin (BSA), 10^−4^ M 2-mercaptoethanol, and 100 ng/mL recombinant mouse (rm) stem cell factor (SCF) (E-Biosciences, USA), 50 ng/mL rm Interleukin-3 (IL-3) (BD-Biosciences, USA) and 50 ng/mL rm granulocyte-macrophage colony stimulating factor (GM-CSF) (BD-Biosciences, USA). The cultures were incubated at 37°C in an atmosphere of 5% CO_2_ in air. At different time course the cultures were monitored and cellular growth pattern was observed and photographed under the inverted microscope for both the normal and experimental groups.

### 2.5. Sample Preparation and Scanning Electron Microscopy

A small portion of the intact marrow tissue from each of the normal control and experimental aplastic groups were kept in 2.8% gluteraldehyde overnight for fixation. To dry the tissue it was repeatedly passed through 30%, 50%, 70%, and 100% gradients of alcohol and finally critical point drying was done. Then, after coating with gold (Au) in IB-2 ion coater, the coated samples went through scanning electron microscopic examination using S-5330 Hitachi SEM and the photographs were recorded.

### 2.6. Bone Marrow Adherent Cell Colony Formation

For the bone marrow adherent cell colony formation, the bone marrow-derived cells were suspended in RPMI-1640 media at a concentration of 4 × 10^6^ cells/plate, supplemented with 30% fetal bovine serum (FBS), 100 U/mL penicillin, 100 U/mL streptomycin and 0.01% (v/v) 2-mercaptoethanol. Four 75 mm petridishes each for the experimental and control groups were plated with 4 mL of media containing 4 × 10^6^ cells and placed in a CO_2_ incubator at 37°C and 5% CO_2_. At every 72 hours interval, the media was drained off and fresh media supplemented with 30% FBS and 0.01% (v/v) 2-mercaptoethanol added for the maintenance of the culture. After 14th, 19th, and 25th days of culture the numbers of colonies were scored using an inverted microscope. 

### 2.7. Hematopoietic Colony Assays

Hematopoietic progenitor cells were assayed in methylcellulose-based semisolid cultures. The culture medium consisted of 1.2% methylcellulose, 30% FBS, 1% BSA, 10^−4^ M 2-mercaptoethanol, and 100 ng/mL recombinant mouse (rm) stem cell factor (SCF), 50 ng/mL rm Interleukin-3 (IL-3) and 50 ng/mL rm granulocyte-macrophage colony stimulating factor (GM-CSF). RBC-depleted bone marrow cells were plated at a final concentration of 5 × 10^5^ cells/mL in triplicate and the cultures were incubated at 37°C in an atmosphere of 5% CO_2_ in air. After 16 days of culture, colonies were scored in the same dish using an inverted microscope. Hematopoietic colonies were classified as follows: CFU-E, erythroid colonies containing 25–50 hemoglobinized cells; BFU-E, erythroid colonies containing more than 50 hemoglobinized cells grouped in one or several clusters. Myeloid colonies comprised the identifiable subpopulations of pure granulocytic colonies (CFU-G), colonies containing both granulocytes and macrophages (CFU-GM). Mixed type of colonies containing both the erythroid and myeloid cells (CFU-GEMM) and the nonhematopoietic colonies of adherent fibroblastic cells (CFU-F) were also found.

### 2.8. Flow Cytometric Analyses of Cell Surface Marker Expression

Flow cytometry was used to detect the expression of combination of markers that are associated with Hematopoietic stem cells (HSCs) (Sca1^+^c-kit^+^) and stromal progenitor cells (Sca1^+^CD44^+^) [38] under normal and diseased condition. Bone marrow cells were washed and fixed with 3% paraformaldehyde (PFA). Then briefly, in one tube 1 × 10^6^, PFA fixed cells were treated with 2 *μ*L each of mouse anti-Sca1 PE and anti-c-kit FITC conjugated monoclonal antibodies (mAbs) (BD-Biosciences, USA). In another tube, 1 × 10^6^ PFA fixed cells were treated with 2 *μ*L each of mouse anti-Sca1 PE and anti-CD44 FITC (BD-Biosciences, USA) conjugated mAbs. All the tubes were incubated in dark at 37°C for 30 minutes. Finally, the cells were washed in PBS and analyzed by BD FACS calibur (Becton-Dickenson, USA), using cell quest pro software (v9.1 Becton Dickenson, USA).

### 2.9. Bone Marrow Cellular Apoptosis Study

Two different aspects of the bone marrow cellular apoptosis profile were monitored in two sets of experiment. The following two approaches were used: (1) expression level of the extracellular apoptosis marker like CD95/APO-1 on bone marrow cells from the normal control as well as the experimental aplastic groups and (2) expression levels of the intracellular apoptosis inducer proteins like pASK-1, pJNK, and Cleaved Caspase-3 on the bone marrow cells from the normal control as well as the experimental aplastic groups.

- For the first protocol, briefly 1 × 10^6^ PFA fixed bone marrow cells were incubated with 2 *μ*L of mouse anti CD95 PE conjugated mAb (BD-Biosciences, USA) in dark at 37°C for 30 minutes. Finally, the cells were washed and analyzed by BD FACS calibur (Becton-Dickenson, USA), using cell quest pro software (v9.1 Becton Dickenson, USA).
- For the intracellular pASK-1, pJNK and cleaved caspase-3 analysis approximately 3 × 10^6^ bone marrow-derived cells were mixed with 1.5% paraformaldehyde. Cells were incubated in fixative for 10 min at room temperature and pelleted. They were then permeabilized by resuspending with vigorous vortexing in 500 *μ*L ice-cold methanol per 1 × 10^6^ cells and incubated at 4°C for 15–20 minutes. Cells were then washed twice in staining media (PBS containing 1% BSA) and divided in three different sorting tubes containing staining media at concentration of 1 × 10^6^ cells per 100 *μ*L. Then accordingly, 2 *μ*L of rabbit anti-mouse pASK-1, pJNK, and cleaved caspase-3 primary mAbs (Cell Signaling Technologies, USA) were added to the respective labeled tubes and incubated for 30 minutes at room temperature followed by the addition of goat anti rabbit secondary antibody conjugated with Alexa Fluor-488 (Invitrogen, USA) to each of primary antibody containing tubes and incubated further for 30 minutes at room temperature. The cells were then washed with 15 volumes of staining media and pelleted. Finally, the pellets, were resuspended in respective tubes containing 100 *μ*L of staining media each and analyzed by BD FACS calibur (Becton-Dickenson, USA), using cell quest pro software (v9.1 Becton Dickenson, USA).

### 2.10. Statistical Analysis

Statistical analysis was performed by using Student's *t*-test, with *P* < .05 accepted as statistically significant.

## 3. Results

### 3.1. Blood Hemogram Profile

In order to confirm the progressive clinical status of the disease in the experimental AA group of mice, blood hemogram profile including hemoglobin concentration, white blood cell (WBC) count, red blood cell (RBC) count, neutrophils, platelets and reticulocytes counts were determined. The results showed a moderate to severe depression in the blood hemoglobin concentration in AA (11.14 g/dl) compared to the normal (15.99 g/dl). Total blood cell corpuscular counts were also severely reduced with uniform reduction in the reticulocyte count in AA (0.22%) compared to the normal (0.89%). Total WBC, RBC, and neutrophils counts were also decreased in the experimental AA group of mice (WBC-3.1 × 10^3^/*μ*L, RBC-4.2 × 10^6^/*μ*L, Neutrophils-9.95%) compared to their normal counterparts (WBC-6.2 × 10^3^/*μ*L, RBC-8.44 × 10^6^/*μ*L, Neutrophils-22.75%).

### 3.2. Long-Term Bone Marrow Culture

**Figure 1:**
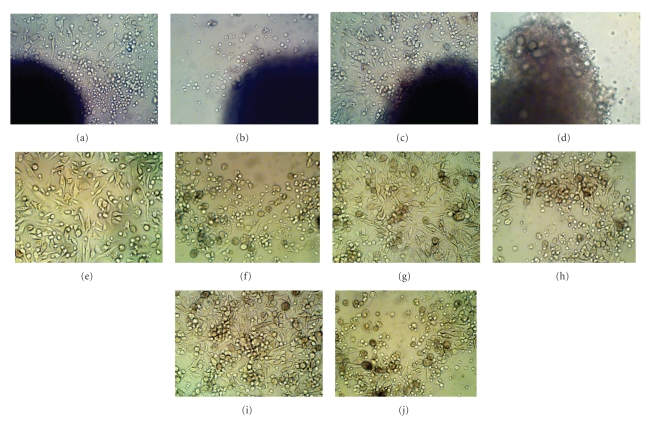
(a) The explant from the normal bone marrow showed healthy cell generation at day 3 of culture. (b) Explant from the aplastic bone marrow showed stunted cellular growth with little or no cellular generation at day 3 of culture. (c) At day 7 of culture, explant from the normal bone marrow demonstrated a cumulative cell production with the efficacy of stromal matrix formation which was completely absent in the explant from aplastic marrow (d). (e) After 11 days of culture, the cells released from the normal bone marrow created various stromal microenvironments like structures throughout the culture dish distal from the explant compared to the aplastic bone marrow where no such stromal matrix generation was observed with predominant appearance of immature stromal precursors and stromal fibroblasts (f). (g) After day 16 of culture, the cells from the normal bone marrow produced a coordinated network of mature stromal matrix of fibroblastic cells upon which small hematopoietic cellular colony formation can be noticed. (h) After 16 days of the culture, the cells from the aplastic bone marrow failed to generate a functional stromal matrix that can sustain hematopoiesis with abundance of immature giant stromal precursors and fibroblastic cells. (i) After 19 days of culture, the normal marrow generated stromal matrix was appeared to be even more organized with appearance of large hematopoietic colonies associated with it, compared to the aplastic marrow where no such functional stromal matrix formation with associated cellular colony generation was observed (j).

To analyze the marrow hematopoietic microenvironment and specific microenvironmental components that are essential for maintaining normal hematopoiesis, long-term bone marrow explant cultures (LTBMC-Ex) provide the systematic model to demonstrate normal bone marrow microenvironmental associations and its alterations in the bone marrow failure diseases like AA. The normal bone marrow explant showed healthy cell generation at day 3 of culture (Figure 1(a)) compared to the aplastic marrow that showed stunted cell generation pattern (Figure 1(b)). After 7 days of culture, the normal explant showed efficiency of stromal matrix formation (Figure 1(c)) compared to that of AA where the explant appeared to be distorted and fragile with almost no cellular generation (Figure 1(d)). At day 11 of culture, the cells released from the normal bone marrow created stromal matrix throughout the different areas of the culture dish distal from the explant (Figure 1(e)) compared to the AA bone marrow that showed no such stromal matrix generation with predominant appearance of immature stromal precursors and stromal fibroblasts (Figure 1(f)). At day 16 of the culture, the cells from the normal bone marrow generated an organized network of mature stromal matrix of fibroblastic cells upon which small hematopoietic colony formation can be noticed (Figure 1(g)) in comparison with the cells form the AA bone marrow that failed to generate a functionally mature stromal matrix that can sustain hematopoiesis (Figure 1(h)). After 19 days of culture, the normal marrow generated stromal matrix appeared to be even more coordinated with appearance of large hematopoietic colonies associated with it (Figure 1(i)), compared to the AA marrow where no such functional stromal matrix formation was observed with frequent appearance of large immature stromal precursors and stromal fibroblasts (Figure 1(j)).

### 3.3. Scanning Electron Micrographs of Bone Marrow Ultrastructure

**Figure 2:**
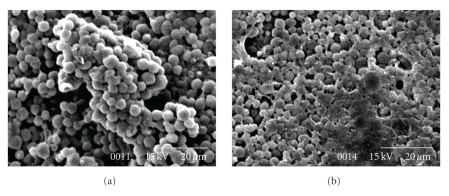
(a) The scanning electron micrographs of the normal bone marrow represented a well-organized compact cellular structures and intracellular association between the stem and stroma in the normal marrow microenvironment. (b) Scanning electron micrographs from the AA bone marrow acquainted an overall scanty and degenerative marrow with no such intertwined stem-stroma structure and distorted cellular components with frequent appearance of apoptic holes.

Scanning electron micrographs (SEM) of the normal and AA bone marrow exhibited convincing differences in their microenvironmental ultrastructure. The scanning electron micrographs of the normal bone marrow (Figure 2(a)) represented a well-organized compact cellular structure and intracellular association between the stem and stroma in the normal marrow microenvironment. Scanning electron micrographs from the AA bone marrow (Figure 2(b)) showed an overall scanty and degenerative marrow with no such intertwined stem-stroma structure and distorted cellular components with frequent appearance of apoptic holes.

### 3.4. Bone Marrow Adherent Cell Colony

**Figure 3:**
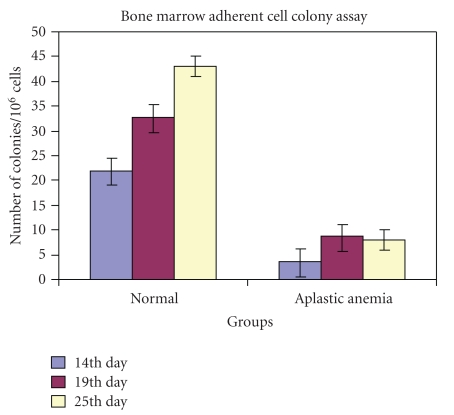
The quantitative distribution chart of the bone marrow adherent cell colony numbers at different day's interval at 14th, 19th, and 25th days of culture from the normal and aplastic anemic groups of mice (*n* = 15) showed an average decrease in adherent cell colony numbers in aplastic anemia through out the duration of culture compared to that of the normal (*n* = 15) (*P* < .02).

The quantitative distribution chart of the bone marrow adherent cell colony numbers (Figure 3) displayed that colony forming ability of the bone marrow adherent cell population from the aplastic group of mice was always low in number compared to that of the normal control group throughout the days of culture. The quantitative distribution chart showed that after day 14 and 19 of culture, the experimental AA groups of animals had mean values only 15% and 33% of the control mean (*P* < .02), respectively. After day 25 of culture, the colony numbers still decreased and the mean value for AA was found to be only 21% of the control mean (*P* < .02). The overall mean value of adherent bone marrow cell colony numbers in AA was only 26% of the control mean (*P* < .02).

### 3.5. Colony-Forming Assay

**Figure 4:**
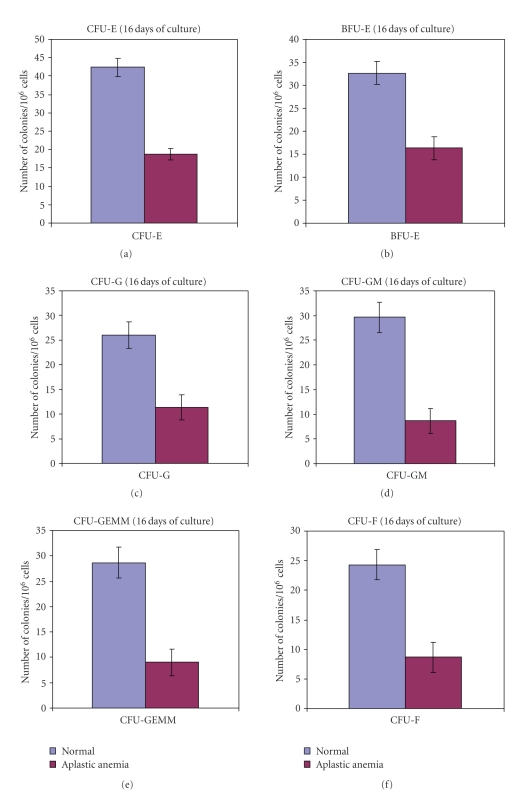
Colony forming efficiency of the bone marrow-derived cells from the normal and aplastic anemic groups of mice. (a) Colony forming unit erythrocytes (CFU-E) from the normal and aplastic bone marrow cells. (b) Erythroid burst forming unit (BFU-E) in normal and AA. (c) Colony forming unit granulocytes (CFU-G) by the AA and normal bone marrow cells. (d) Colony forming unit granulocytes macrophages (CFU-GM) from the AA and normal bone marrow cells. (e) Colony forming unit granulocyte erythrocyte monocyte macrophage (CFU-GEMM) in normal and AA. (f) Colony forming unit fibroblasts form the normal and AA bone marrow cells. All the results represent the relative number of CFUs in aplastic anemia as compared to the normal and correspond to the median ± S.D. levels observed from 15 (*n* = 15) samples studied (*P* < .01).

Colony-forming efficiency of the bone marrow derived cells from the normal and aplastic anemic groups of mice depicted an overall decrease in colony-formation by the AA bone marrow progenitor cells in comparison with the normal marrow progenitors. Colony-forming unit erythrocytes (CFU-E) (Figure 4(a)), erythroid burst forming unit (BFU-E) (Figure 4(b)) showed an overall decrease in AA that indicated a significant (*P* < .01) decrease in mature erythrocyte formation. Colony forming unit granulocytes (CFU-G) (Figure 4(c)), colony forming unit granulocytes macrophages (CFU-GM) (Figure 4(d)), colony forming unit granulocyte erythrocyte monocyte macrophage (CFU-GEMM) (Figure 4(e)) documented a moderate to severe (*P* < .01) decrease in AA indicated a dysfunction of the marrow stem/progenitor cells in terms of their ability to multilineage differentiation in AA. Colony forming unit fibroblasts (CFU-F) also showed a decrease (*P* < .01) in AA with respect to the normal value (Figure 4(f)) which was well correlated with the deficient stromal function observed through long-term marrow culture. All the results represented the relative number of CFUs in aplastic anemia as compared to the normal and corresponded to the median ± S.D. levels observed from 15 (*n* = 15) samples studied (*P* < .01).

### 3.6. Flow Cytometric Analysis of Cell Surface Receptor Expression

**Figure 5:**
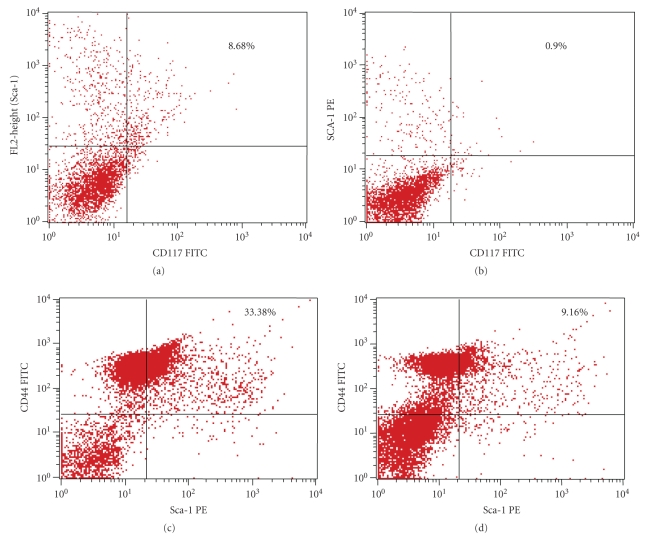
Phenotypical characterization of Sca1 and c-kit (CD-117) receptor expression by the bone marrow cells from normal (a) and Aplastic Anemic group (b) showed a quantitative depression in the primitive stem cell population in aplastic anemia. Phenotypical characterization of stromal progenitor cell marker (Sca1 and CD44) expression by the normal (c) and aplastic (d) bone marrow cells depicted depletion in the stromal progenitor cell population in aplastic anemia.

Phenotypic characterization of Sca1 and c-kit receptor expression by bone marrow primitive stem cells indicated a highly downregulated Sca1^+^c-kit^+^ cell population in the AA group of animals (0.90%) (Figure 5(b)) compared to the normal controls (8.68%) (Figure 5(a)).

Further, the phenotypic characterization of the bone marrow-derived stromal progenitor (Sca1^+^CD44^+^) cells demonstrated a depressed stromal progenitor population in the AA group (9.16%) (Figure 5(d)) compared to the normal (33.38%) (Figure 5(c)). The results documented a significant downregulation in both the stem and stromal progenitor cell population in AA bone marrow which was highly correlated with what we have observed through long-term bone marrow culture and colony forming efficacy of the bone marrow-derived progenitors in AA.

### 3.7. Cellular Apoptosis Study

Bone marrow cellular apoptosis profile was monitored by means of two different aspects.

**Figure 6:**
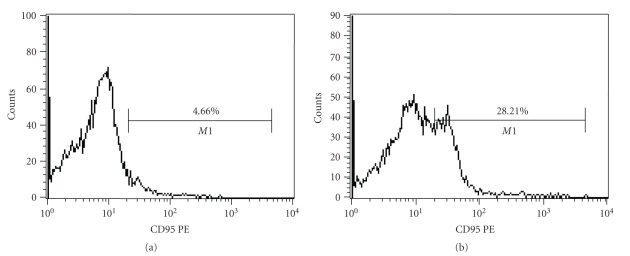
(a) CD95 expression by the normal bone marrow cells. (b) Increased CD95 expression on the bone marrow cells in aplastic anemia showed a significant high level of apoptosis in the bone marrow cell population in aplastic anemia.

**Figure 7:**
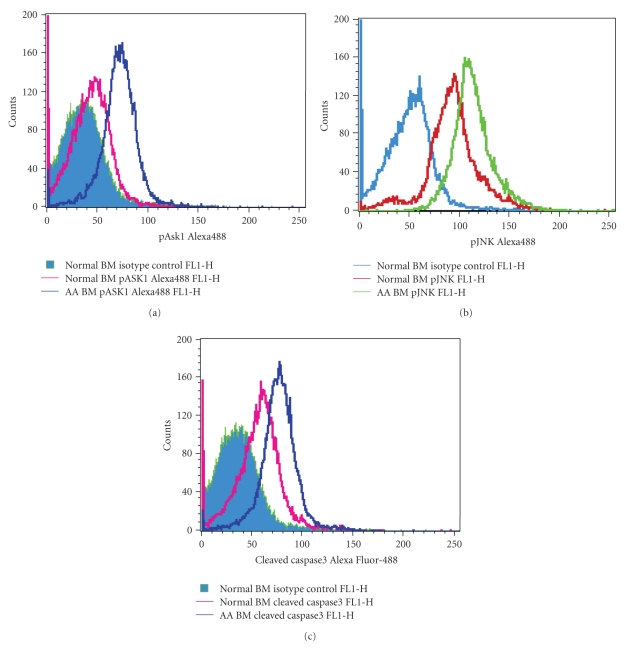
Flowcytometric analysis of intracellular apoptosis inducer proteins levels in normal and aplastic bone marrow cells. (a) Aplastic bone marrow cells (blue line) labeled with p-ASK1 showed a significant increase in median value of fluorescence intensity compared to the normal (red line). (b) Aplastic bone marrow cells (green line) labeled with phospho-JNK documented a significant increase in median value of fluorescence intensity in comparison with the normal (red line). (c) Aplastic bone marrow cells (blue line) labeled with cleaved caspase-3 documented convincing increase in median value of fluorescence intensity as compared to the normal (red line).

- High extracellular CD95 expression was evidenced on the bone marrow-derived cells of AA group (28.21%) (Figure 6(b)) compared to the normal (4.66%) (Figure 6(a)) that indicated an enhanced bone marrow cellular apoptosis level in AA which was well correlated with the decreased stem and stromal progenitor population in AA.
- Flow cytometric analysis of intracellular apoptosis inducer protein levels in normal and aplastic bone marrow cells showed an increased intracellular apoptotic protein expression levels in AA compared to the normal. Data was presented here as median value of fluorescence intensity along the *X*-axis. Aplastic bone marrow cells showed a significant increase in intracellular p-ASK1 (Figure 7(a)), p-JNK (Figure 7(b)) and Cleaved Caspase-3 (Figure 7(c)) levels as compared with the normal marrow cells. The results represented an increased intracellular apoptosis inducer protein expression pattern in aplastic anemia which was significantly correlated with our previous data of increased Fas-R (CD-95) expression on the AA bone marrow cells.

## 4. Discussion

The pathogenesis of AA still remains obscure. Although several other studies suggested that the microenvironmental defects might have play a role in the progression of bone marrow failure in AA, but the exact mechanism was not clear. We have used long-term bone marrow explant culture as a useful tool to assess the stromal microenvironmental defect in AA. Long-term bone marrow culture represents the most physiologic system for assessing growth and proliferation of the bone marrow cells in vitro. As the proliferation kinetics of the hematopoietic bone marrow cells are dependent on the complex interaction between the hematopoietic cells and a confluent stroma, so our system to study stroma and assess the functional capacity of the stromal components represented a more physiological in-vitro monitoring system. In this paper, we have primarily assessed the stromal (adherent) functional efficacy and the hematopoietic ability of the AA bone marrow cells through long-term marrow culture and colony forming efficacy of the bone marrow-derived cells, respectively. In culture, the normal bone marrow explant showed healthy cellular generation pattern with subsequent development of a functional stromal matrix that could sustain hematopoiesis in the long term. In sharp contrast to the normal scenario, the AA bone marrow explant documented a stunted cell growth characteristic with almost no sign of formation of a functional stromal adherent layer. However, 20% of AA bone marrow explants exhibited a moderate marrow stromal adherent layer formation but that also declined rapidly with days of progression of culture with concurrent decrease in the number of nonadherent and committed progenitor cells than did the normal controls that implied a defect in either the stem cells level or in the capacity of marrow stromal cells to support hematopoiesis. Using long-term bone marrow explant culture we demonstrated a defect in the regenerative capacity of not only the hematopoietic cells but also the supportive stroma. Our result clearly showed a defective proliferative capacity of the AA bone marrow cells in culture. The defective hematopoiesis may have resulted from either the failure of the stromal microenvironment to produce growth factors essential for hematopoiesis or the absence of proper growth factor receptors on the AA bone marrow cells. 

Bone marrow ultrastructural analysis depicted a degenerative marrow compartment in AA. The composite normal cellular organization was totally absent in AA bone marrow with frequent abundance of apoptic pits within it. The structural integrity of the bone marrow microenvironment which is essential for maintaining normal hematopoiesis was found to be totally lost in AA. The disorganized marrow microenvironment hinted at the “microenvironment dependent degeneration of normal hematopoiesis” in AA.

Our study demonstrated a significantly lowered level of colony formation by the adherent (stromal) bone marrow cell population in AA. This data constituted another piece of evidence for the existence of stromal defect in AA. Taking into consideration the leading role of stromal cells in regulation of early HSC function, the stromal cell dysfunction may be critical for inhibition of hematopoiesis in AA. 

 Inhibitory effects of AA hematopoietic microenvironment were also evidenced with significantly low CFUs forming ability by the marrow hematopoietic stem/progenitor cells in AA. The decreased values of CFU-E, BFU-E, CFU-G, CFU-GM, and CFU-GEMM essentially pointed towards a residual defect in the marrow hematopoietic stem/progenitor cell compartment in terms of clonogenic potential in AA. The defect could have originated either from the hematopoietic stem/progenitor cell level or from the nonhematopoietic stromal cell level depending on their physiological crosstalk in the hematopoietic microenvironment. The extended discussion of this fact might include two fundamental points: (1) a functionally impotent stroma-dependent inhibition of hematopoietic stem/progenitor cell proliferation and (2) an impairment in the physiological maturation and cell surface receptor expression in the hematopoietic stem/progenitor cells that are essential for maintenance of the cells in the hematopoietic microenvironment. 

**Figure 8:**
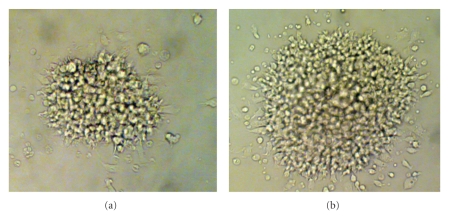
(a) Colony Forming Unit-Fibroblast (CFU-F) in Normal. (b) Colony Forming Unit-Fibroblast (CFU-F) in Aplastic Anemia.

Functionally abnormal fibroblasts have been documented in AA. The marrow fibroblasts showed defective production of fibroblast colony forming unit (CFU-F) in AA. The decreased CFU-F further hinted at the defective microenvironmental stromal fibroblast function. However, the size and concentration of CFU-F was found to be increased in AA (Figure 8(b)) compared to the normal (Figure 8(a)). This paradoxical data might account for the marrow compensatory mechanism and result of additional mitosis due to the activation of stromal precursor cells to support the mesenchymal cells under stressed marrow condition in AA.

The flow cytometric analysis of bone marrow cells represented a quantitative loss in both the stem (Sca-1^+^c-kit^+^) and stromal (Sca-1^+^CD44^+^) progenitor population in AA. Though the study represented here mainly focused on co-evaluation of the defects of intertwined microenvironmental components, yet we have specifically quantified the isolated bone marrow stem and stromal progenitor cells as these components are designated the most essential and integral units of the hematopoietic microenvironment. The sharp decrease in the stem and stromal progenitor population provided the required supportive base to explain the actual event of deranged hematopoiesis in AA. 

Apoptosis or programmed cell death (PCD) is the most discussed pathophysiologic mechanism associated with the aplastic disease progression. The pathophysiology of apoptosis has only recently been identified, but there is substantial evidence that disturbance of mechanisms controlling cell death may play an important role in the diseases associated with abnormal cell death and/or cellular proliferation. The relationship between the apoptosis and the aplastic disease progression is reversible; that is, the increased bone marrow cellular apoptosis leads to the increased occurrences of the disease aplastic anemia. In this present paper, we wanted to throw light on some apoptosis regulators (both the extracellular transmembrane receptor protein (CD-95) and the intracellular apoptosis inducer proteins like p-ASK-1, p-JNK, and cleaved caspase-3) that played a major role in controlling normal cellular apoptosis and their subsequent alterations in AA. The aplastic anemic bone marrow cells showed an increased transmembrane CD95 (Fas-R) expression. The increased Fas-R expression resulted in higher rate of apoptosis in AA. The interpretation of the increased expression of Fas-R on the AA bone marrow cells might include two points: either (1) there was an increment in the levels of inhibitory factors secreted by the stromal microenvironment that potentially induce apoptosis by acting as ligands to Fas-R or (2) there was an immune-mediated destruction of marrow cells by contact-mediated transmembrane signaling that has been shown to involve the surface molecule Fas-R (CD95) on target bone marrow cells and its ligands on cytotoxic T lymphocytes (CTLs). The increased FasR-FasL interaction then potentially switched on the apoptosis signaling pathway by activating (phosphorylating) several intracellular apoptosis inducer proteins such as ASK-1, JNK, and caspase-3 in a stage specific manner. In our study, we have specifically analyzed these activated forms of intracellular proteins that is, pASK-1, pJNK, and cleaved caspase-3, and we have obtained an increased level of all the activated proteins in the bone marrow cells in AA. The result obtained has helped us to understand the apoptosis pathway involved in AA (Figure 9). 

In conclusion, we can say that the AA bone marrow documented alterations with respect to certain components of the stromal microenvironment, including the defect in ability to support normal hematopoiesis, adherent cell colony formation and CFU-F formation. Simultaneously, the stem cell population also seemed to be severely affected in aplastic anemia with decreased clonogenic potential as evident from CFU formation and flow cytometric analysis. Long-term bone marrow culture revealed the inhibitory effects of the stromal microenvironment that were responsible for the proliferative defects in the hematopoietic cells. The alterations in the stromal microenvironment possibly accounted most for the pathogenesis of AA. Accordingly, we suggest that further investigations into the stromal microenvironment and the stromal components in AA would be necessary for better understanding the mechanism of the disease.

## Figures and Tables

**Figure 9:**